# Pathway analysis of smoking-induced changes in buccal mucosal gene expression

**DOI:** 10.1186/s43042-022-00268-y

**Published:** 2022-03-17

**Authors:** Anas Khaleel, Bayan Alkhawaja, Talal Salem Al-Qaisi, Lubna Alshalabi, Amneh H. Tarkhan

**Affiliations:** 1 Department of Pharmacology and Biomedical Sciences, Faculty of Pharmacy and Medical Sciences, University of Petra, Amman, Jordan; 2 Department of Pharmaceutical Medicinal Chemistry and Pharmacognosy, Faculty of Pharmacy and Medical Sciences, University of Petra, Amman, Jordan; 3 Department of Medical Laboratory Sciences, Pharmacological and Diagnostic Research Centre, Faculty of Allied Medical Sciences, Al-Ahliyya Amman University, Amman, Jordan; 4 Independent Researcher, Doha, Qatar

**Keywords:** Gene expression, Cigarette smoking, Ingenuity Pathway Analysis, Buccal mucosa

## Abstract

**Background:**

Cigarette smoking is the leading preventable cause of death worldwide, and it is the most common cause of oral cancers. This study aims to provide a deeper understanding of the molecular pathways in the oral cavity that are altered by exposure to cigarette smoke.

**Methods:**

The gene expression dataset (accession number GSE8987, GPL96) of buccal mucosa samples from smokers (*n* = 5) and never smokers (*n* = 5) was downloaded from The National Center for Biotechnology Information's (NCBI) Gene Expression Omnibus (GEO) repository. Differential expression was ascertained via NCBI’s GEO2R software, and Ingenuity Pathway Analysis (IPA) software was used to perform a pathway analysis.

**Results:**

A total of 459 genes were found to be significantly differentially expressed in smoker buccal mucosa (*p*  < 0.05). A total of 261 genes were over-expressed while 198 genes were under-expressed. The top canonical pathways predicted by IPA were nitric oxide and reactive oxygen production at macrophages, macrophages/fibroblasts and endothelial cells in rheumatoid arthritis, and thyroid cancer pathways. The IPA upstream analysis predicted that the TP53, APP, SMAD3, and TNF proteins as well as dexamethasone drug would be top transcriptional regulators.

**Conclusions:**

IPA highlighted critical pathways of carcinogenesis, mainly nitric oxide and reactive oxygen production at macrophages, and confirmed widespread injury in the buccal mucosa due to exposure to cigarette smoke. Our findings suggest that cigarette smoking significantly impacts gene pathways in the buccal mucosa and may highlight potential targets for treating the effects of cigarette smoking.

**Supplementary Information:**

The online version contains supplementary material available at 10.1186/s43042-022-00268-y.

## Background

Tobacco smoking is responsible for one in six of all deaths from non-communicable diseases, leading experts to identify tobacco control as the highest priority public health intervention [1, 2]. The prevalence of smoking has fallen around the world over the past three decades, but the absolute number of people who smoke has increased [3]. Despite a coordinated worldwide effort against smoking, there are around 1.1 billion current smokers, and it is expected that this number would reach 1.9 billion by 2025 if current smoking patterns are maintained [4].

Cigarette smoke contains over 5000 chemicals, of which 98 have been identified as carcinogenic or probably carcinogenic to humans [5]. The plethora of carcinogens in cigarette smoke perturbs biological pathways related to cellular proliferation, inflammation, and tissue injury, with strong links to various types of cancer [6, 7]. In cancer patients, cigarette smoking has been associated with an increased symptom burden as well as a reduced efficacy of chemotherapy [6, 8].

Smoking-induced differential gene expression has been well-documented in previous studies. In fact, smoking has a characteristic impact on the transcriptome, as it activates inflammatory and oxidative responses, changes airway structures, and alters gene expression across tissue types [9]. Previous studies have shown that cigarette smoking significantly alters the gene expression profiles of adipose tissue, buccal cells, nasal epithelial cells, lung tissue, and whole blood [10–14].

The aim of the current study is to broaden the understanding of the molecular pathways that are altered in buccal mucosa after exposure to cigarette smoke. Gene expression data from smokers and never smokers were analyzed via Ingenuity Pathway Analysis (IPA), which is a web-based software application that identifies new targets within the context of biological systems.

## Methods

### Data acquisition

The microarray dataset investigated in the present study was obtained from The National Center for Biotechnology Information’s (NCBI) Gene Expression Omnibus (GEO) repository (accession number GSE8987). This dataset included gene expression data of buccal mucosa samples from smokers (*n* = 5) and never smokers (*n* = 5) [15]. Smokers were classified as those who had smoked at least 10 cigarettes per day and who had a cumulative smoking history of at least 10 pack years [15]. Table 1 shows the gene expression data samples included in the current study.

**Table 1:** Gene expression data samples included in the current analysis

Sample nos.	Subject	Type of sample	Source of sample
GSM227858	Never smoker	RNA	Buccal mucosa
GSM227859	Never smoker	RNA	Buccal mucosa
GSM227860	Never smoker	RNA	Buccal mucosa
GSM227861	Smoker	RNA	Buccal mucosa
GSM227862	Smoker	RNA	Buccal mucosa
GSM227863	Smoker	RNA	Buccal mucosa
GSM227864	Smoker	RNA	Buccal mucosa
GSM227865	Smoker	RNA	Buccal mucosa
GSM227866	Never smoker	RNA	Buccal mucosa
GSM227867	Never smoker	RNA	Buccal mucosa

As per the original study by Sridhar et al., buccal mucosa samples were collected from the study participants by scraping the inside of their mouths with a concave plastic tool with serrated edges. Total RNA was extracted from buccal mucosa samples using TRIzol reagent (Invitrogen, Carlsbad, CA), and RNA integrity was assessed using a denaturing agarose gel. The Affymetrix Human Genome U133A (HG-U133A) Array (Affymetrix, Santa Clara, CA) was then used to profile the gene expression of the extracted total RNA samples [15].

The demographics of the 10 subjects varied with regard to sex, age, and race. Among the 5 smokers, the mean age was 36 years old (± 8 years), with 1 male and 4 females. Similarly, the mean age of the 5 never smokers was 31 years old (± 9 years), with 2 males and 3 females. In terms of race, the smoker group comprised 3 Caucasians and 2 African Americans, while the never-smoker group consisted of 2 Caucasians and 3 African Americans. Demographic data for individual subjects were not provided in the dataset, but statistical comparisons of the smoker and never-smoker groups revealed not significant *p* values for sex (*p* = 0.42), age (*p* = 0.36), and race (*p* = 0.40) [15].

### Identification of differentially expressed (DE) genes

The GEO2R software, which is available on the NCBI website, was used to create a list of 15,000 differentially expressed genes between smoker and never-smoker buccal samples.

The 15,000 genes were inputted into a Microsoft Excel spreadsheet and sorted by significance (Additional file 1: Table S1). After applying strict cut-off criteria (*p* < 0.05 and absolute fold change between − 0.5 and 1.5), the list of DE genes was narrowed down to 459 genes.

The Bioconductor package Enhanced Volcano was used to visualize the 459 DE genes in the form of a labelled volcano plot [16].

### Ingenuity pathway analysis (IPA)

The list of DE genes was inputted into IPA software (QIAGEN, Hilden, Germany), where the ‘core analysis’ function of the software was used to interpret the data in terms of canonical pathways and upstream regulators.

### Pathway and functional enrichment analysis

The Bioconductor package clusterProfiler was used to carry out an over-representation analysis of the DE genes [17, 18]. Similarly, the SIGnaling Network Open Resource 2.0 (SIGNOR 2.0) was used to explore the signaling networks that exist between the DE genes [19].

## Results

### Differentially expressed (DE) genes

**Fig. 1:**
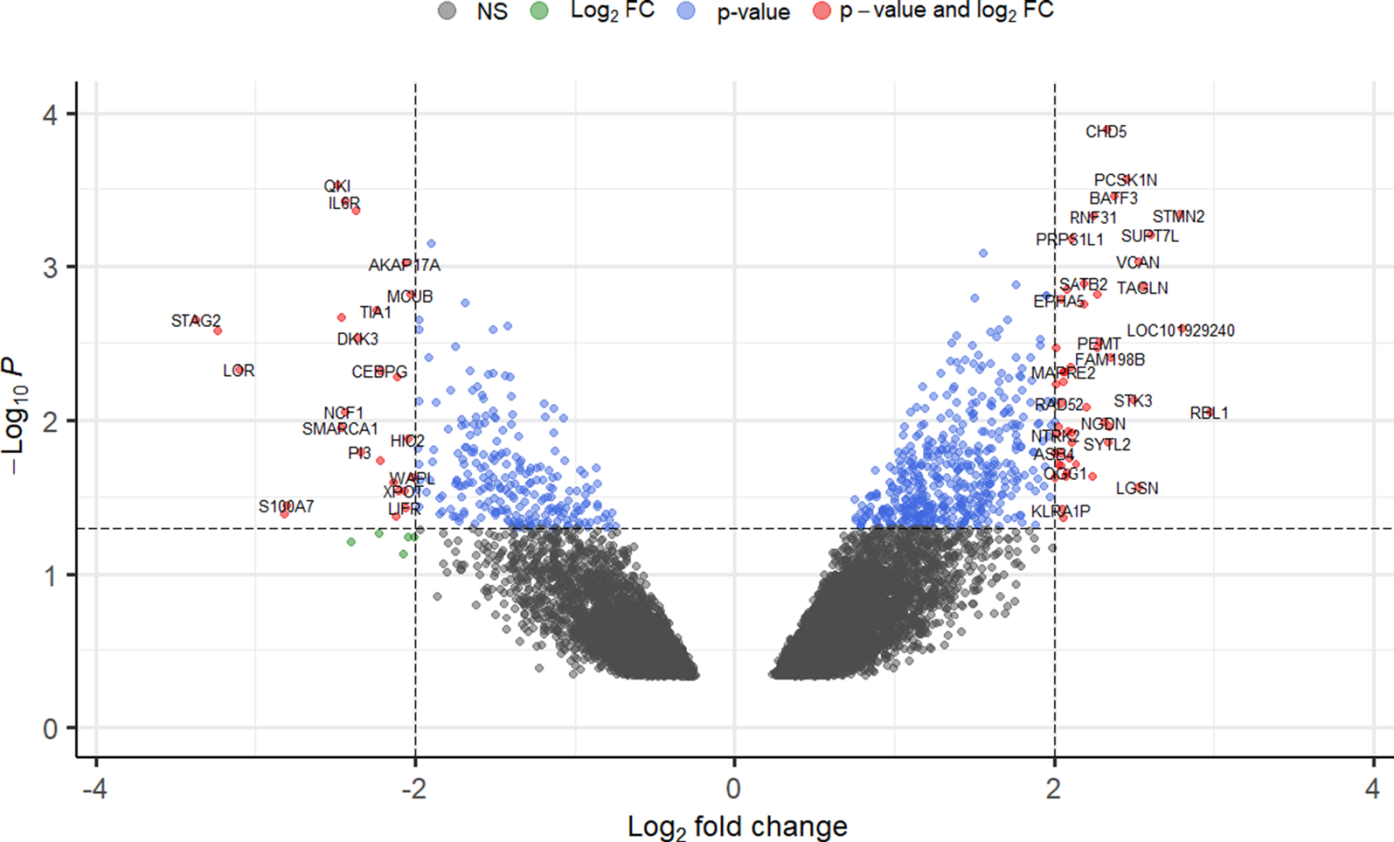
Volcano plot of the most significantly differentially expressed genes in smoker buccal mucosa. Gray points are non-significant, green points have a significant log2FC, blue points have a significant *p* value, and red points have both a significant log2FC and *p* value

Figure 1 displays a volcano plot of the full list of DE genes. However, only 459 genes exhibited significant differential expression, with 261 genes found to be over-expressed and 198 found to be under-expressed.

**Fig. 2:**
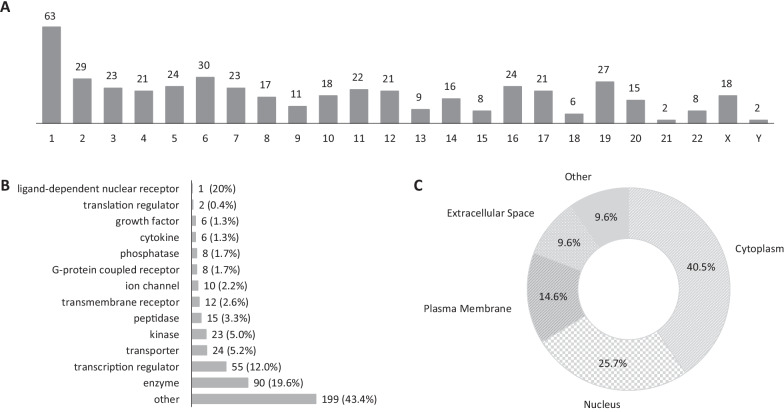
Distribution of most significantly differentially expressed genes in terms of **A** chromosomal location, **B** molecular class, and **C** cellular location

Figure 2 illustrates the chromosomal location, molecular class, and cellular location of the 459 DE genes. Chromosome 1 had the highest number of significantly DE genes (*n* = 63), followed by chromosome 6 (*n* = 30), chromosome 2 (*n* = 29), and chromosome 19 (*n* = 27). Similarly, the most represented molecular classes among the significantly DE genes were enzymes (19.6%) and transcription regulators (12%). Lastly, the majority of the significantly DE genes were located either in the cytoplasm (40.5%) or the nucleus (25.7%).

Table 2 lists the most significantly DE genes between smoker and never smoker buccal mucosa samples, showing that protein-coding genes occupy the top ranks in terms of significance.

**Table 2:** Significantly differentially expressed genes in smokers as revealed by IPA and as sorted by *p* value

NCBI ID	Gene symbol	Gene name	Chr	Molecule type	Expression	Log_2_ ratio	*p* value
*Top differentially expressed protein-coding genes*							
26038	*CHD5*	Chromodomain helicase DNA binding protein 5	1	Enzyme	Up	2.335	1.28 × 10^–04^
27344	*PCSK1N*	Proprotein convertase subtilisin/kexin type 1 inhibitor	X	Other	Up	2.45	2.72 × 10^–04^
9444	*QKI*	QKI, KH domain containing RNA binding	6	Other	Down	− 2.484	2.97 × 10^–04^
55509	*BATF3*	Basic leucine zipper ATF-like transcription factor 3	1	Transcription regulator	Up	2.379	3.54 × 10^–04^
3570	*IL6R*	Interleukin 6 receptor	1	Transmembrane receptor	Down	− 2.444	3.80 × 10^–04^
51302	*CYP39A1*	Cytochrome P450 family 39 subfamily A member 1	6	Enzyme	Down	− 2.371	4.35 × 10^–04^
11075	*STMN2*	stathmin 2	8	Other	Up	2.786	4.65 × 10^–04^
55072	*RNF31*	Ring finger protein 31	14	Enzyme	Up	2.249	4.74 × 10^–04^
9913	*SUPT7L*	SPT7 like, STAGA complex subunit gamma	2	Transcription regulator	Up	2.605	6.26 × 10^–04^
221823	*PRPS1L1*	Phosphoribosyl pyrophosphate synthetase 1 like 1	7	Kinase	Up	2.102	6.64 × 10^–04^
*Top differentially expressed lncRNA genes*							
100505933	*ADD3-AS1*	ADD3 antisense RNA 1	10	Other	Up	2.075	1.42 × 10^–03^
6315	*ATXN8OS*	ATXN8 opposite strand lncRNA	13	Other	Up	1.952	7.57 × 10^–03^
100506070	*RBFADN*	RBFA downstream neighbor	18	Other	Up	1.683	9.10 × 10^–03^
100131532	*LOC100131532*	Uncharacterized LOC100131532	6	Other	Up	2.136	1.93 × 10^–02^
25859	*PART1*	Prostate androgen-regulated transcript 1	5	Other	Down	− 0.942	3.01 × 10^–02^
100130449	*LOC100130449*	Uncharacterized LOC100130449	2	Other	Down	− 1.603	3.52 × 10^–02^
*Top differentially expressed pseudogenes*							
388714	*FMO6P*	Flavin containing dimethylaniline monoxygenase 6, pseudogene	1	Other	Up	1.778	5.11 × 10^–03^
442240	*ZNF259P1*	Zinc finger protein 259 pseudogene 1	6	Other	Up	2.054	5.65 × 10^–03^
228	*ALDOAP2*	ALDOA pseudogene 2	10	Other	Down	− 1.573	1.05 × 10^–02^
79986	*ZNF702P*	Zinc finger protein 702, pseudogene	19	Other	Up	1.653	1.32 × 10^–02^
645,682	*POU5F1P4*	POU class 5 homeobox 1 pseudogene 4	1	Other	Up	1.521	1.46 × 10^–02^
5408	*PNLIPRP2*	Pancreatic lipase related protein 2 (gene/pseudogene)	10	Enzyme	Down	− 1.437	1.61 × 10^–02^
440915	*POTEKP*	POTE ankyrin domain family member K, pseudogene	2	Other	Up	1.62	1.93 × 10^–02^
730092	*RRN3P1*	RRN3 pseudogene 1	16	Other	Up	1.548	1.96 × 10^–02^
150244	*ZDHHC8P1*	ZDHHC8 pseudogene 1	22	Other	Up	1.559	2.27 × 10^–02^
202181	*LOC202181*	SUMO interacting motifs containing 1 pseudogene	5	Other	Up	1.932	2.67 × 10^–02^

*Chr*, chromosome; log_2_ ratio, log_2_ fold change ratio of the gene expression between 2 groups

### Interaction network of differentially expressed (DE) genes

**Fig. 3:**
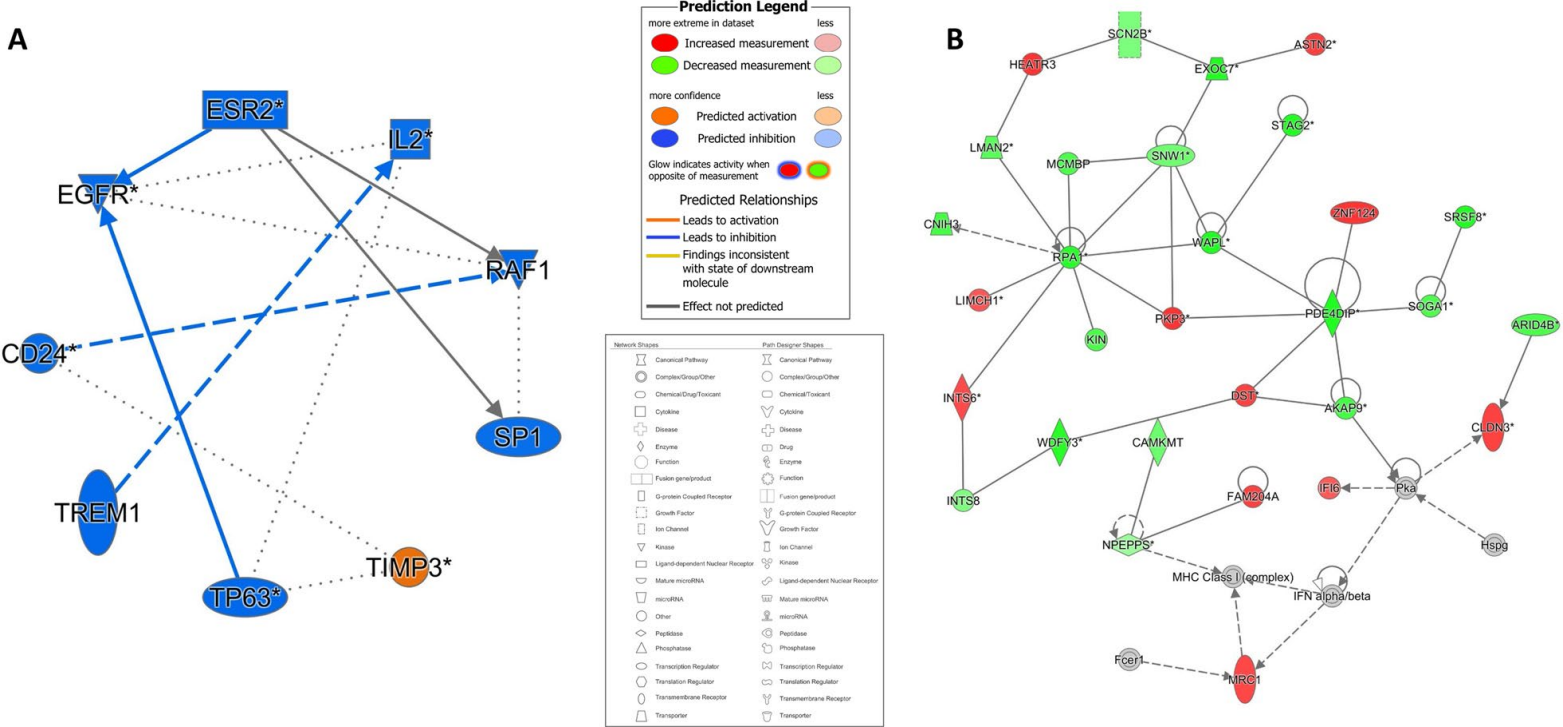
**A** Graphical summary and **B** interaction network analysis of differentially expressed genes in smoker buccal mucosa. Different shapes represent the molecular class of the protein. Red and green indicate upregulation and downregulation, respectively, while blue and orange indicate inhibition and activation, respectively. A solid line indicates a direct interaction, a dashed line indicates an indirect interaction, and a dotted line indicates inferred correlation from machine-based learning. An asterisk indicates that multiple identifiers in the dataset file map to a single gene or chemical in the Global Molecular Network

Figure 3A demonstrates the interplay between the DE oncological pathways, cytokines, and genes in smoker buccal mucosa, namely the *IL2*, *EGFR*, and *ESR2* genes. Other than TIMP3, all the proteins in the pathway were predicted to be inhibited in smoker buccal mucosa.

Figure 3B illustrates the results of an interaction network analysis of the DE genes in smoker buccal mucosa. Interestingly, the *RPA1* gene was shown to have the highest number of interactions with the other DE genes in smoker buccal mucosa, but it did not have a significant level of differential expression (*p* > 0.05).

### Upstream regulators

**Fig. 4:**
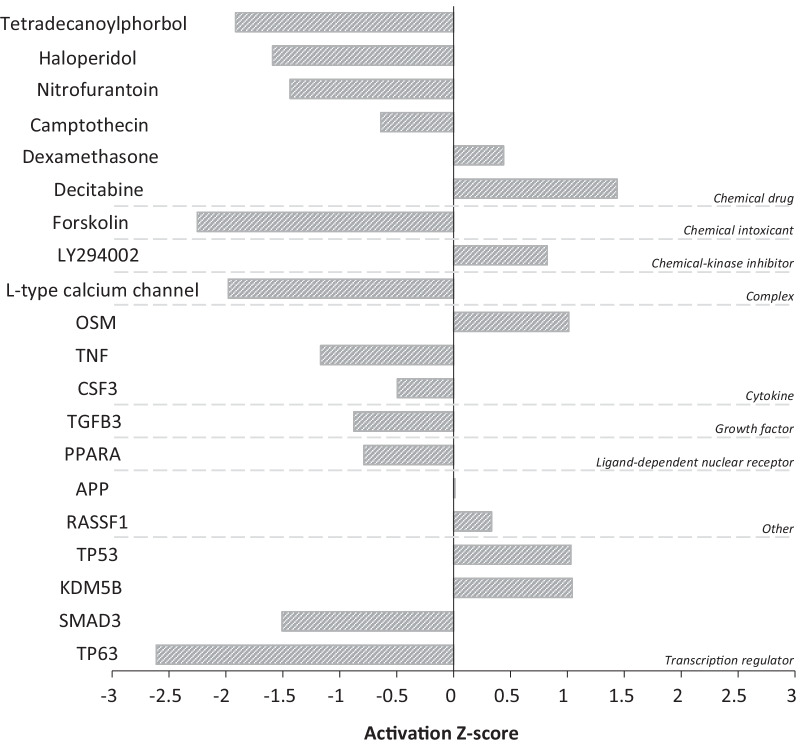
Top upstream regulators predicted by Ingenuity Pathway Analysis in smoker buccal mucosa. A positive z score indicates activation, while a negative z score indicates inhibition

The top 20 regulators predicted by IPA included the TP53, APP, SMAD3, and TNF proteins as well as the drug dexamethasone, among other molecules (Table 3). Figure 4 illustrates the data in Table 3 and emphasizes the predicted activation status of the top upstream regulators as revealed by IPA. As can be seen from Fig. 4, the most inhibited upstream regulator in smoker buccal mucosa is predicted to be the TP63 protein.

**Table 3:** Top 20 upstream regulators revealed by Ingenuity Pathway Analysis

Upstream regulator	Molecule type	Log_2_ ratio	*p* value	Z score
TP53	Transcription regulator	0.477	1.39 × 10^–09^	1.028
Dexamethasone	Chemical drug	–	1.10 × 10^–06^	0.438
APP	Other	1.709	1.39 × 10^–06^	0.01
SMAD3	Transcription regulator	0.687	3.20 × 10^–06^	− 1.508
TNF	Cytokine	0.098	5.90 × 10^–06^	− 1.169
Decitabine	Chemical drug	–	6.61 × 10^–06^	1.433
Tetradecanoylphorbol	Chemical drug	–	8.14 × 10^–06^	− 1.916
L-type calcium channel	Complex	–	1.01 × 10^–05^	− 1.98
Camptothecin	Chemical drug	–	1.53 × 10^–05^	− 0.64
KDM5B	Transcription regulator	0.95	1.79 × 10^–05^	1.04
OSM	Cytokine	− 0.844	2.74 × 10^–05^	1.01
TGFB3	Growth factor	0.488	2.80 × 10^–05^	− 0.877
Nitrofurantoin	Chemical drug	–	2.90 × 10^–05^	− 1.437
Forskolin	Chemical toxicant	–	4.11 × 10^–05^	− 2.25
TP63	Transcription regulator	1.346	4.15 × 10^–05^	− 2.611
CSF3	Cytokine	0.301	6.00 × 10^–05^	− 0.494
PPARA	Ligand-dependent nuclear receptor	0.147	6.01 × 10^–05^	− 0.789
Haloperidol	Chemical drug	–	6.35 × 10^–05^	− 1.591
LY294002	Chemical-kinase inhibitor	–	7.53 × 10^–05^	0.822
RASSF1	Other	0.33	8.26 × 10^–05^	0.333

**Fig. 5:**
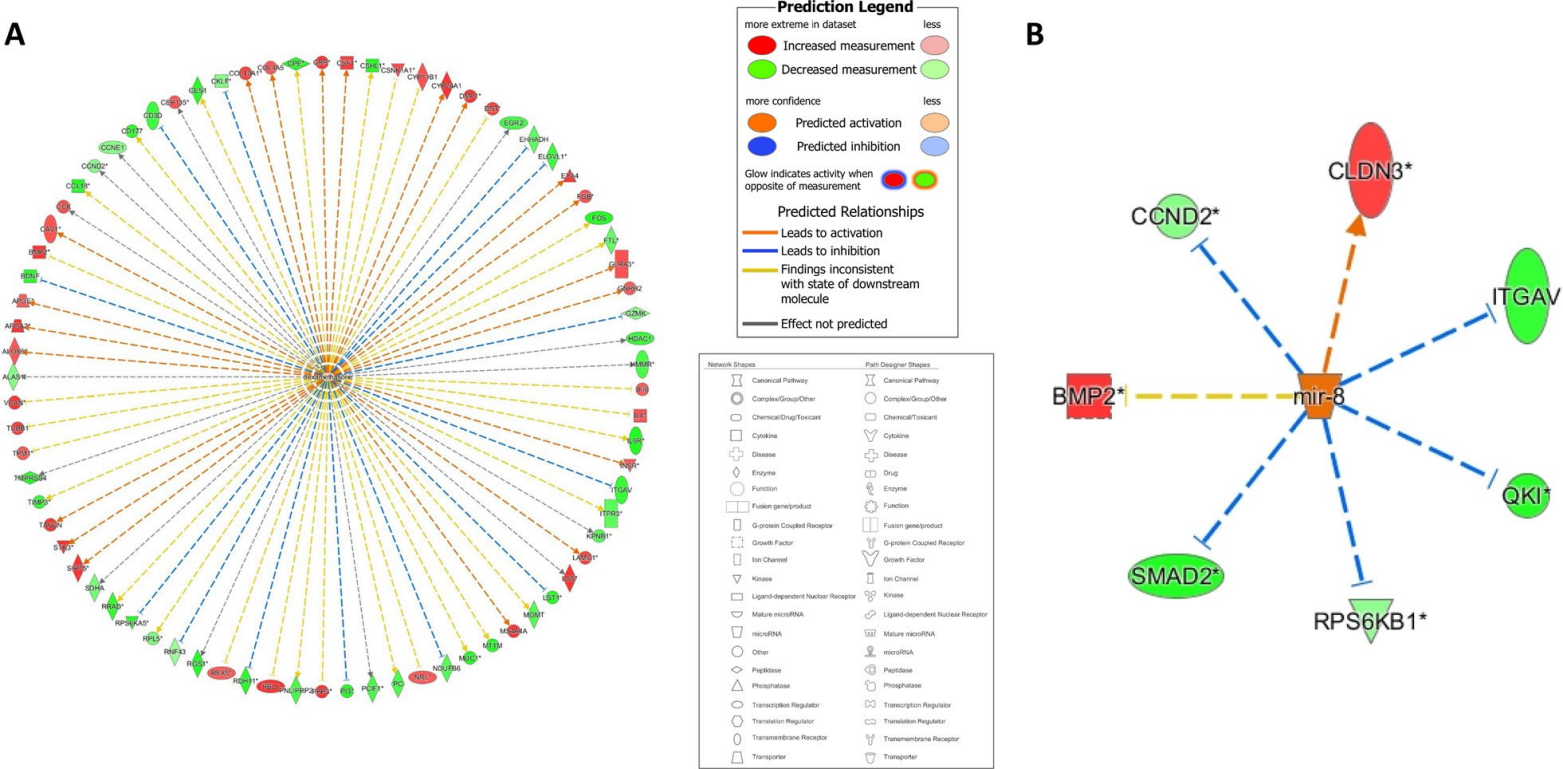
Upstream regulators dexamethasone and miR-8. **A** Chemical drug dexamethasone is predicted to be activated in smoker buccal mucosa with P value = 1.10 × 10^–06^ and Z score = 0.438. **B** miR-8 is predicted to be activated in smoker buccal mucosa with *P* value = 8.90 × 10^–02^ and Z score = 2.1. Different shapes represent the molecular class of the protein. Red and green indicate upregulation and downregulation, respectively, while blue and orange indicate inhibition and activation, respectively. A solid line indicates a direct interaction, a dashed line indicates an indirect interaction, and a dotted line indicates inferred correlation from machine-based learning. An asterisk indicates that multiple identifiers in the dataset file map to a single gene or chemical in the Global Molecular Network

Dexamethasone was predicted to be a top upstream regulator and affected a total of 78 genes via indirect interactions (Fig. 5A). Likewise, microRNA-8 (miR-8) was found by IPA to be among the top upstream regulators to be activated, as miR-8 targeted 7 of the DE genes between smokers and never smokers (Fig. 5B). Of those genes, 5 (*CCND2*, *ITGAV*, *QKI*, *RPS6KB1*, and *SMAD2*) were under-expressed and 2 (*BMP2* and *CLDN3*) were over-expressed.

**Fig. 6:**
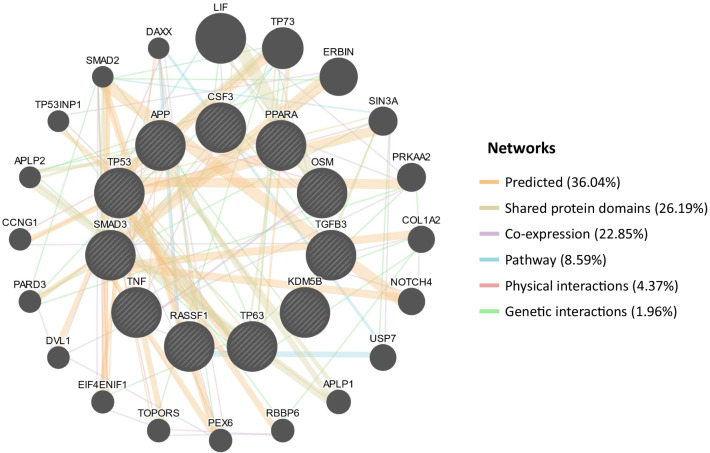
Construction of a gene–gene interaction network of the upstream regulators with the most significant differential expression. Black circles with white stripes indicate genes that were entered as query terms, while black circles indicate the associated genes. The size of the circle corresponds with the number of correlations with other genes in the network

**Fig. 7:**
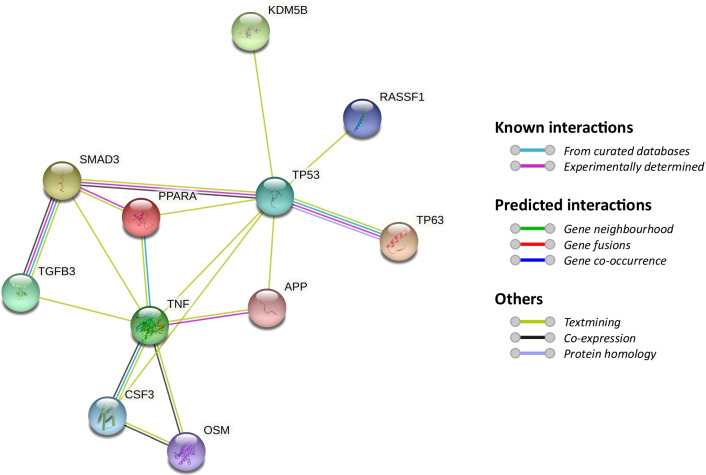
Protein–protein interaction network of the top upstream regulators with the most significant DE

Further analysis of the top upstream regulator proteins resulted in the construction of gene–gene (Fig. 6) and protein–protein (Fig. 7) interaction networks. Figure 6 shows that the 36.04% of the top upstream regulator proteins were predicted to have interactions with one another, 26.19% have shared protein domains, and 22.85% were co-expressed. Similarly, Fig. 7 shows that the TP53 and TNF proteins had the highest number of interactions with the other top upstream regulator proteins.

### Enriched biological pathways

The most significant canonical pathway was identified as the nitric oxide and reactive oxygen production at macrophages (Table 4).

**Table 4:** Top five canonical pathways revealed by ingenuity pathway analysis

Pathway	*p* value	− log(*p* value)	Z score
Nitric oxide and reactive oxygen production at macrophages	0.00124	2.9	− 1.26
Retinol biosynthesis pathways	0.00159	2.7	− 1.3
Thyroid tumor signaling pathways	0.00441	2.3	− 0.81
Insulin signaling pathways	0.00860	2.0	− 2.11
Macrophages/fibroblasts and endothelial cells in rheumatoid arthritis	0.00895	2.0	N/A

### Correlation of smoker buccal mucosa with other diseases

The DE genes in smoker buccal mucosa are significantly associated with cancer and organismal injury, among other diseases (Table 5).

**Table 5:** Top five diseases or disorders revealed by Ingenuity Pathway Analysis

Disease	Number of molecules	*p* value
Organismal injury/abnormalities	436	5.00 × 10^–04^ to 6.75 × 10^–23^
Cancer	432	5.00 × 10^–04^ to 6.75 × 10^–23^
Gastrointestinal disease	393	4.66 × 10^–04^ to 1.01 × 10^–14^
Endocrine system disorders	385	4.99 × 10^–04^ to 1.25 × 10^–18^
Reproductive system disease	311	4.99 × 10^–04^ to 7.24 × 10^–13^

### Pathway and functional enrichment analysis

**Fig. 8:**
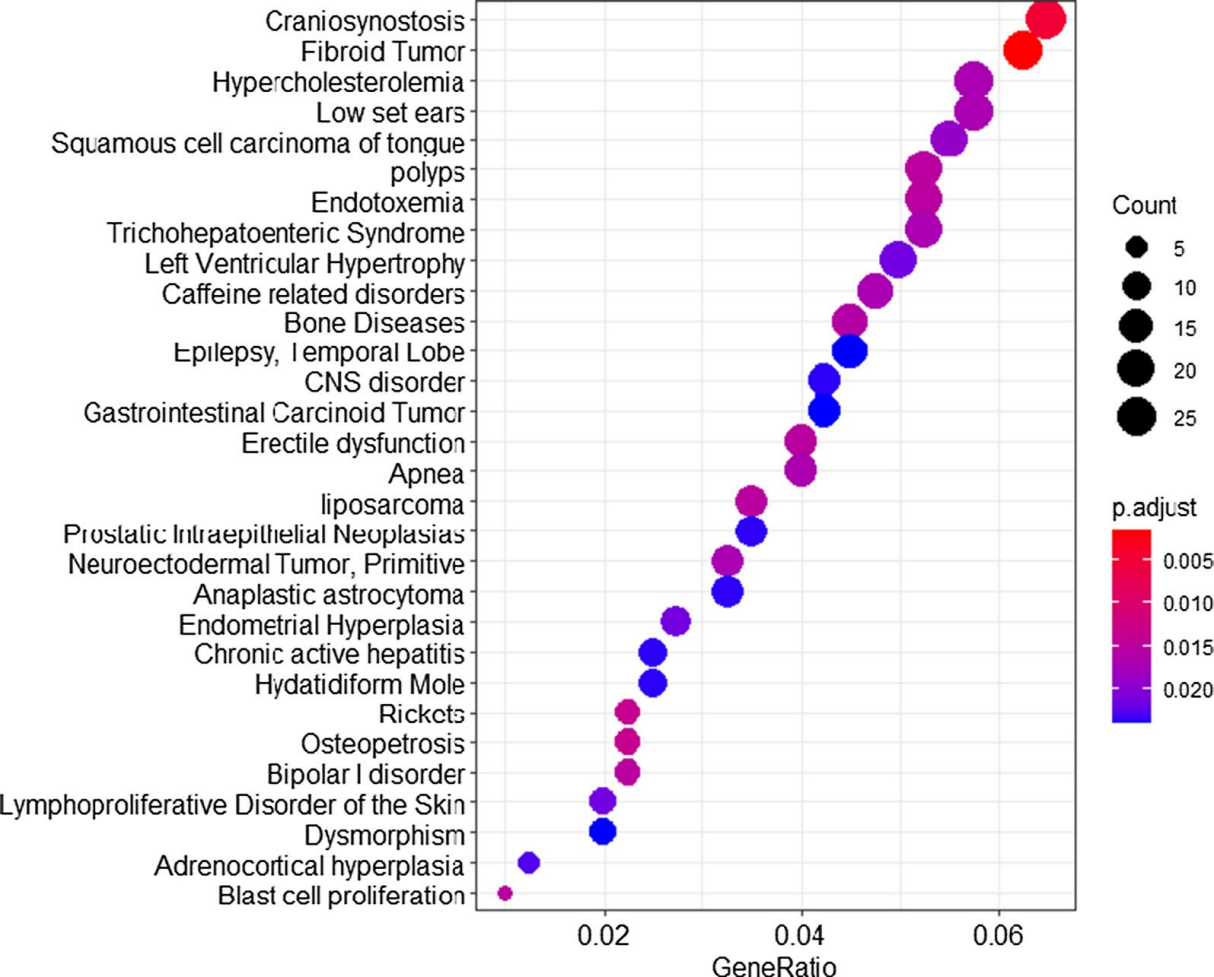
Over-representation analysis of the most significantly differentially expressed genes (*p* < 0.05). The size of the circle corresponds with the number of genes that are mapped to a biological process, while a redder color indicates increased significance

Figure 8 illustrates the most over-represented biological processes in smoker buccal mucosa. Interestingly, craniosynostosis and fibroid tumors were revealed to be the topmost significantly over-represented biological processes.

**Fig. 9:**
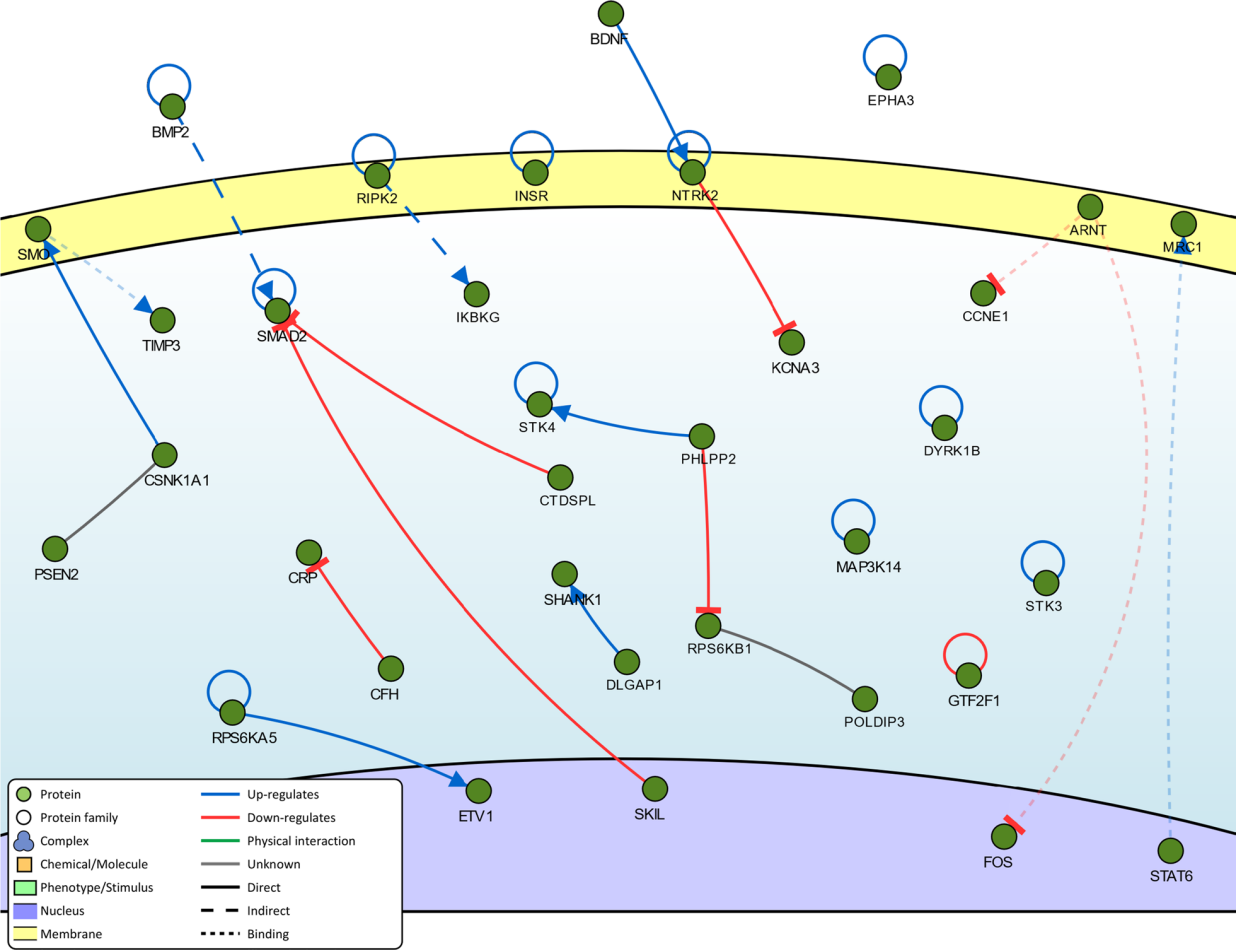
Pathway signaling network generated from the most significantly differentially expressed genes. Different shapes represent molecular class, while red and blue indicate downregulation and upregulation, respectively

Figure 9 shows the results of signaling network analysis of the 459 significantly DE genes, with the *SMAD2* gene having the most interactions. *SMAD2* is directly downregulated by the *CTDSPL* and *SKIL* genes and indirectly upregulated by the *BMP2* gene.

## Discussion

The most significantly differentially expressed (DE) protein-coding genes in smoker buccal mucosa were the *CHD5*, *QKI*, *BATF3,* and *IL6R* genes, which have previously reported associations with smoking and related diseases.

The *CHD5* gene, which is a tumor suppressor gene that is preferentially expressed in the nervous system and testis, was significantly upregulated in smoker buccal mucosa [20, 21]. *CHD5* is believed to serve as a master regulator in tumor-suppressive networks, and *CHD5* expression levels are strongly associated with the prognosis of several cancers, including hepatocellular carcinoma and non-small cell lung cancer [20, 22–24]. One study found that a rare *CHD5* variant, rs12564469-rs9434711, contributed to the risk of hepatocellular carcinoma, a risk effect which was statistically significant in alcohol drinkers but not smokers [25].

The *QKI* gene contributes to a number of human diseases, including cancers, myelin disorders, and schizophrenia, and it is a critical regulator of alternative splicing in cardiac myofibrillogenesis and contractile function [26]. *QKI* has also been identified as a master regulator of alternative splicing in human lung cancer cell lines, but no significant statistical association was found between *QKI* expression and smoking status in lung tumors [27, 28]. Moreover, *QKI* was identified as a significantly altered gene in the ciliated epithelial cells of lungs affected by chronic obstructive pulmonary disease (COPD), a disease that is primarily caused by tobacco smoking [29].

The *BATF3* gene belongs to the AP-1 transcription factor family, whose members respond to a range of pathological and physiological stimuli by mediating gene expression [30]. BATF3 controls the differentiation of dendritic cells, inhibits the differentiation of regulatory T cells, and critically regulates the development of memory T cells [31, 32]. *BATF3* expression in the lungs was necessary in order to induce protection against allergic airway inflammation through tolerization with *Helicobacter pylori* extract [33]. Moreover, the acute inhalation of electronic cigarette smoke by healthy never smokers led to the significant upregulation of *BATF3*, among other genes that play a role in promoting tumorigenesis [34].

The *IL6R* gene is a pleiotropic regulator of both acquired and innate immune responses, and it is believed to be expressed in the lungs [35]. There have been conflicting findings regarding the benefits of anti-IL-6R therapy for COVID-19-induced acute respiratory distress syndrome [36, 37]. In the context of smoking, exposure to cigarette smoke led to increased *IL6R* mRNA levels in primary bronchial epithelial cell lines [38]. Moreover, a certain *IL6R* haplotype (rs6684439-rs7549250-rs4129267-rs10752641-rs407239) has been associated with a lower COPD risk in a Mexican Mestizo population, while the IL6R variant Asp358Ala did not show any association with COPD [39, 40].

Pseudogene expression was also altered in smoker buccal mucosa, most notably in the upregulation of *FMO6P*, *ZNF259P1*, and *ZNF702P* and the downregulation of *ALDOAP2* and *PNLIPRP2*. *FMO6P* has significant sequence homology with the *FMO3* gene, the latter of which functions to metabolize a small amount of nicotine [41]. A single nucleotide variation in the *FMO6P* pseudogene, rs6608453, was associated with nicotine dependence in African Americans [42]. Likewise, *ALDOAP2* was over-expressed in both healthy and non-healthy smokers compared to non-smokers, while exposure to cigarette smoke resulted in the upregulation of the *PNLIPRP2* polymorphic pseudogene in a murine model [43, 44]. In contrast, *ZNF259P1* and *ZNF702P* did not have previously reported associations with smoking. *ZNF259P1* was significantly correlated with the tumor size of primary lung adenocarcinomas, while *ZNF702P* was found to be upregulated after *BCL2L10* knockdown in two ovarian cell lines [45, 46].

Analysis of upstream regulators revealed that the tumor protein 53 (*TP53*) gene was the most significantly DE regulator in smoker buccal mucosa. *TP53* contains cellular proliferation by guarding against genomic mutation, and *TP53* mutations are among the most common genetic alterations in human cancers [47]. Tobacco smoking is known to influence *TP53* mutation patterns and frequencies in lung cancer and urothelial cell carcinoma patients [48, 49]. In fact, a large proportion of *TP53* mutations in the lung cancers of smokers were G → T transversions, a primary mutagenic signature that is caused by DNA damage from tobacco smoke [50].

The most significant canonical pathway identified by IPA was the “nitric oxide and reactive oxygen production at macrophages”. Nitric oxide and reactive oxygen species are essential for maintaining redox balance, but they also act in pathological processes [51]. Tobacco smoke contains large numbers of free radicals, including nitric oxide and reactive oxygen species (ROS), that cause oxidative stress on the cellular and sub-cellular levels [52, 53]. In turn, smoking-induced oxidative stress activates inflammatory response pathways that produce endogenous ROS at the site of oxidative stress, potentially causing further oxidative damage to that site [53]. Smoking also reduces the production of nitric oxide while also elevating the production of ROS in endothelial cells [54, 55]. Smoking-induced ROS production is especially concerning as it may contribute to the progression of endometrial adenocarcinoma [56].

Among the DE genes, those associated with craniosynostosis and fibroid tumors were over-represented in smoker buccal mucosa.

Craniosynostosis, which is caused by the premature fusion of cranial sutures, is the second-most common cranio-facial anomaly [57]. Smoking during pregnancy was associated with an increased risk of craniosynostosis, while exposure to secondhand smoke modestly increased the risk of this birth defect [58]. Maternal smoking impacts cranio-facial development by acting upon variant alleles of the transforming growth factor alpha (TGF-α) gene, and genetic variation of the TGF-α gene is associated with increased risk of cranio-facial defects [59, 60].

Fibroid tumors are non-cancerous growths that develop inside or on the uterus and are the most common type of pelvic tumor detected in women [61]. Previous studies that investigated the impact of smoking on fibroid tumors yielded conflicting results. Earlier studies suggested that smoking had a protective effect against fibroid tumors, but subsequent studies have shown either a negative effect or no relationship at all [61, 62]. It is worthwhile to note that smoking has been shown to have an anti-estrogenic effect in women, resulting in an earlier natural menopause as well as protective associations with the risk of estrogen-related cancers [63, 64].

Pathway network analysis revealed that the *SMAD2* gene had the highest number of interactions with other DE genes, and it was also a target of miR-8. *SMAD3* was predicted by IPA to be an inhibited upstream regulator. The SMAD Family Member 2 (*SMAD2*) gene encodes for a protein that is vital for early development, and *SMAD2* mutations were associated with complex cranio-facial defects in a murine model [65]. SMAD2, SMAD3, and SMAD4 mediate the signal transduction of transforming growth factor-β (TGF-β) superfamily members, the latter of which induce a range of effects that involve cellular differentiation, proliferation, migration, and apoptosis [66].

The present study is affected by a few limitations. The sample size was relatively small, and the patient samples differed in terms of sex and race, which could confound the interpretation of the genetic variation. Additionally, several differentially expressed genes in smoker buccal mucosa were uncharacterized or unmapped to pathways, meaning that their effects are not considered in the current analysis.

## Conclusion

The current findings signify the importance of inflammatory response and oxidative stress as a major component of smoking-induced tissue injury. Most significantly, nitric oxide-related inflammation stands as one of the canonical pathways underlying genetic and molecular pathways changes coupled with exposure to cigarette smoke. Future lines of research should focus on validating the results of the current study in a larger population to ascertain potential therapeutic targets in the context of smoking-induced damage.

## Supplementary Information

**Additional file 1.** List of DE genes downloaded from GEO2R. A complete list of gene names and their abbreviations are delivered in the supplementary data files.

## Data Availability

The current report utilized a previously published dataset for the analysis. The dataset used in this work was acquired from The National Center for Biotechnology Information’s (NCBI) Gene Expression Omnibus (GEO) depository (accession number GSE8987).

## Abbreviations

Chr: Chromosome
COPD: Chronic obstructive pulmonary disease
COVID-19: Coronavirus disease 2019
DE: Differentially expressed
DNA: Deoxyribonucleic acid
GEO: Gene Expression Omnibus
IPA: Ingenuity Pathway Analysis
NCBI: National Center for Biotechnology Information
RNA: Ribonucleic acid
ROS: Reactive oxygen species
SIGNOR 2.0: SIGnaling Network Open Resource 2.0
